# An *In Vitro* Model of Cartilage Degradation by Chondrocytes in a Three-Dimensional Culture System

**Published:** 2012-12

**Authors:** Katsuhito Nakashima, Kensuke Nakatsuka, Kyoko Yamashita, Kurita Kenichi, Hayakawa Taro

**Affiliations:** 1 *Department of Oral and Maxillofacial Surgery, School of Dentistry, Aichi-Gakuin University, Nagoya, Japan;*; 2 *Department of Biochemistry, School of Dentistry, Aichi-Gakuin University, Nagoya, Japan*

**Keywords:** chondrocytes, matrix, TIMPs, cell culture

## Abstract

**Objective:**

Using the alginate bead three-dimensional culturing method, which is considered to be advantageous for the *in vitro* study of chondrocytes, we confirmed earlier reports concerning the inhibitory effect of TGF-β on IL-1β-induced cartilage destruction and serially evaluated changes in proteinases and their inhibitors in cartilage destruction.

**Methods:**

Chondrocytes were cultured on alginate beads with IL-1β or TGF-β alone or both. The glycosaminoglycan (GAG) concentration in the culture medium was determined by use of the DMMB assay; and the levels of TIMP-1, -2 and proMMP-3 were measured with their respective sandwich EIAs. Sections of the beads were prepared and stained with toluidine blue or anti-TIMP-1 -2, -3 antibodies. The numbers of chondrocytes negative for pericellular proteoglycan staining and TIMP-positive chondrocytes were counted, and positive staining for TIMP-3 in the extracellular matrix was examined. RT-PCR was performed to evaluate the gene expression of TIMP-1, -2, -3, and MMP-3.

**Results:**

The number of TIMP-1(+)chondrocytes, TIMP-1 concentration in the culture medium, and TIMP-1-gene expression all increased maximally as early as 6 hours after IL-1β stimulation, and then gradually decreased. However, the number of cells immunopositive for TIMP-3 increased somewhat later. GAG and proMMP-3 concentrations in the culture medium increased gradually with time. The number of TIMP-3(+)chondrocytes and positive staining for TIMP-3 in the extracellular matrix significantly increased in the TGF-β group compared with the values for the IL-1β group. The proMMP-3 concentration in the culture medium of TGF-β-treated cells was significantly decreased compared with that for the IL-1β-treated ones at all times examined.

**Discussion:**

We suggest that TIMP-1 plays a primary role in the prevention of articular cartilage destruction in its early stage but that TIMP-3 gradually takes over this role. Also, TGF-β was shown to regulate these TIMPs and act as a suppressor of articular cartilage destruction. These results suggest that TIMP-1 and TIMP-3 are closely involved in preventing the progression of joint disorders such as OA.

## INTRODUCTION

Articular cartilage is made up of chondrocytes and a rich extracellular matrix (ECM) that surrounds them. The ECM consists of a wide variety of molecules, but its primary component is type II collagen that forms a proteoglycan called aglycan and fibers. While many functions of cartilage such as impact absorption and friction reduction are facilitated by the ECM, the degradation of ECM components is promoted in joint diseases such as osteoarthritis (OA) and causes functional impairment. Matrix metalloproteinase (MMP) and A disintegrin and metalloproteinase with thrombospondin motifs (ADAMTS-4 and -5; also called aggrecanases-1 and -2) are known to play important roles in the degradation of ECM components (1-3) and these enzymes are induced by inflammatory cytokines, typically interleukin-1β (IL-1β) (4). Inflammatory cytokines are involved in the pathology of OA (5, 6) and cause a loss of cartilage matrix by promoting the expression of proteinases as well as suppressing matrix production by chondrocytes.

Antagonizing these factors, cytokines such as transforming growth factor-β (TGF-β) and proteinase inhibitors such as tissue inhibitor of matrix metalloproteinases (TIMPs) inhibit the destruction of the cartilage matrix (7-9). So far, 4 types of TIMP have been reported. These TIMPs bind with MMP at a molar ratio of 1:1 and inhibit its enzyme activity. Particularly, the actions of TIMP-3 are characteristic in that it is the only TIMP that binds with the ECM (10) and it inhibits aggrecanases as well as MMP (11).

The balance between proteinases and their inhibitors is important for the progression of cartilage destruction, and their production is considered to be regulated and controlled by various cytokines, but their changes in joint disorders remain unknown in many respects. These problems have been approached by a number of studies in which proteinases and their inhibitors were induced with IL-1β or TGF-β (12-16). However, monolayer cultures were used in many of them, and whether the results obtained reflect the original properties of chondrocytes is questionable, because chondrocytes dedifferentiate and transform into fibroblast-like cells in monolayer cultures. To answer this question, there have been attempts to three-dimensionally culture chondrocytes in an environment closer to the *in vivo* state (17, 18). Particularly, the three-dimensional culturing method using alginate beads is advantageous in that it allows the long-term culturing of chondrocytes while maintaining their original properties, and they can be readily isolated and collected by the solation of alginate through EDTA treatment. In these alginate bead three-dimensional cultures, the diversity of lacunae formed by individual chondrocytes under the influence of IL-1β and TGF-β appear to reflect their responses to inflammatory cytokines, and evidence that the lacuna can be regarded as the minimum unit of cartilaginous tissue has been obtained (19).

Under such circumstances, using the alginate bead three-dimensional culturing method, which is considered to be advantageous for the *in vitro* study of chondrocytes, we confirmed earlier reports concerning the inhibitory effect of TGF-β on IL-1β-induced cartilage destruction and serially evaluated changes in proteinases and their inhibitors in cartilage destruction.

## MATERIALS AND METHODS

### Three-dimensional culturing using alginate beads

Tissue sections of articular cartilage were sampled aseptically from the shoulder (proximal articular surface of the humerus) and knee of a young Japanese white rabbit (female, body weight: 1 kg). Chondrocytes were isolated by treating the sampled cartilage sections with 0.4% actinase (Kaken Pharmaceutical) at 37°C for 1 hour, and then with 0.025% collagenase (Roche Diagnostics) at 37ºC for 14 hours. The isolated chondrocytes were suspended in 1.2% alginate physiologic saline at 1 × 10^6^ cells/ml, and this suspension was dripped into a 102 mM CaCl_2_ solution and allowed to form a gel by gently stirring for 10 minutes. After alginate beads were washed 3 times using 10 volumes of 0.15 M NaCl solution, three-dimensional culturing was initiated. Culturing was carried out using Dulbecco’s modified Eagle’s minimal essential medium (D-MEM) supplemented with 10% fetal calf serum (FCS) (Gibco Laboratories) at 37ºC in 5% CO_2_ for 7 days.

### Addition of drugs and recovery of cells and culture medium

After culturing for 7 days, the cells were cultured for 24 hours in D-MEM not containing FCS and stimulated with interleukin-1β (IL-1β) (Roche Diagnostics) at 5 ng/mL or transforming growth factor-b (TGF-β) (Roche Diagnostics) at 10 ng/mL alone or with both agents in combination. Combined stimulation was performed by adding IL-1β after pretreating the cells with TGF-β for 6 hours. The durations of stimulation with a single agent or both agents were 6, 12, 24, 48, 72, or 96 hours.

After stimulation over the various durations, the culture medium only was carefully collected using a syringe to which an 18 G injection needle was attached. Alginate beads were removed for morphological evaluation, and the remaining alginate was dissolved by treating with a lysing solution (55 mM sodium citrate, 0.15 M NaCl, 30 mM EDTA-2Na, pH6.8) at 4ºC for 10 minutes. Centrifugation was performed for 10 minutes, and chondrocytes were recovered by washing the sediment 2 times using phosphate-buffered saline (PBS).

### Evaluation using serial sections of alginate beads

**Figure 1:**
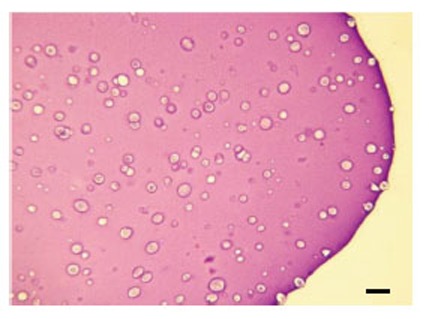
Toluidine blue staining profile of rabbit articular chondrocytes cultured in alginate beads (×250). Lacunae varying widely in morphology are observed. Scale bar = 100 μm.

**Figure 2:**
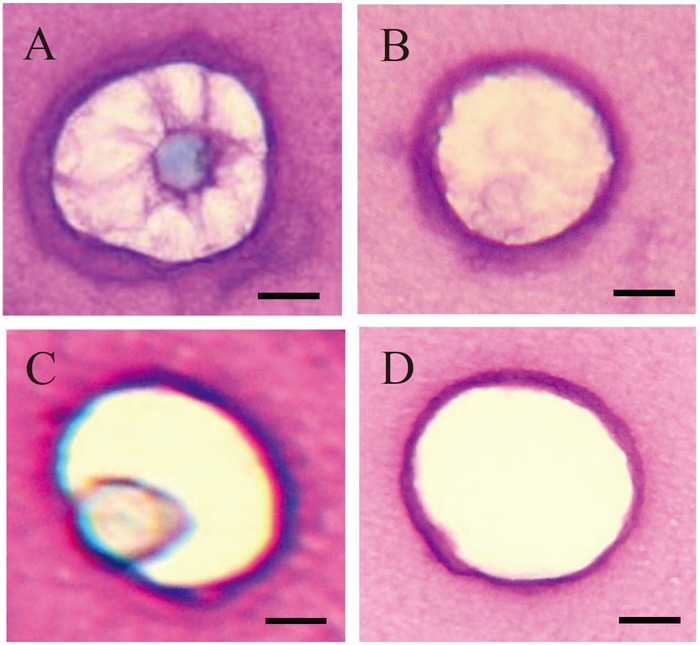
Lacunae varying widely in morphology observed in alginate beads stained with toluidine blue. A: Cells and cellassociated matrix (CAM); B: CAM alone; C: Cells and margins of lacunae; D: Margins of lacunae alone. Of the lacunae with various morphological characteristics, those showing no staining for CAM (C and D) were counted. Scale bar = 10 μm.

**Toluidine blue staining.** Alginate beads for morphological evaluation were fixed for 4 hours using 4% paraformaldehyde (dissolved with 0.1 M cacodylate buffer, pH7.4, containing 10 mM CaCl_2_) and washed overnight with 0.1 M cacodylate buffer (pH7.4, containing 50 mM BaCl_2_). After embedding in paraffin, 4 mm serial sections were prepared and mounted on glass slides. They were first stained with 0.04% toluidine blue-O (SIGMA), pH4.0 (Fig. 1). After staining, of the lacunae observed in alginate bead sections in various experimental groups and at various durations of stimulation (Fig. 2), those with no stained cell-associated matrix (CAM) were counted. Their percentage relative to the total number of lacunae contained in an alginate bead section was calculated. Through this experiment, the extents of destruction by treatment with IL-1β and TGF-β were examined, and the duration of treatment was determined to be until the effect of each cytokine became clear. Also, to calculate the percentage of various TIMP-positive cells described below, the total number of chondrocytes was determined.

**Figure 3:**
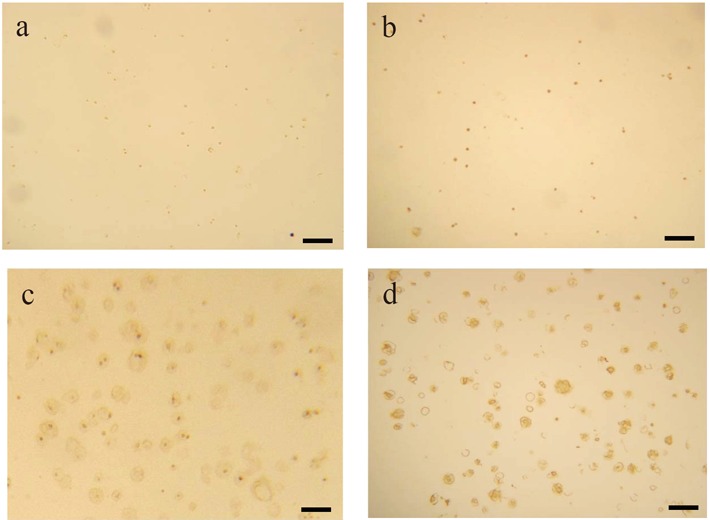
Immunostaining profiles of rabbit articular chondrocytes cultured in alginate beads (×250). a: Anti-TIMP-1 antibody; b: Anti-TIMP-2 antibody; c: Anti-TIMP-3 antibody; d: CS-56. Lacunea varying widely in morphology are observed. Scale bar = 100 μm.

**Immunostaining using anti-TIMP-1, TIMP-2, TIMP-3, and chondroitin sulfate monoclonal antibodies.** The serial sections of alginate beads were then stained immunologically using anti-TIMP-1, TIMP-2, and TIMP-3 monoclonal antibodies (Daiichi Fine Chemical) and anti-chondroitin sulfate monoclonal antibody (CS-56, Seikagaku Corporation), and staining was compared among the experimental groups and according to the duration of treatment. Immunostaining was performed using the ABC method with peroxidase labeling using DAB as a coloring agent (Fig. 3). On immunostaining using anti-TIMP-3 monoclonal antibody and CS-56, the percentage of lacunae with no staining for CAM was evaluated similarly to toluidine blue staining. Also, cells that showed positive immunostaining with anti-TIMP-1, TIMP-2, and TIMP-3 monoclonal antibodies were counted, and their percentages relative to the number of cells determined above by toluidine blue staining were compared. The positive cell rate on each immunostaining was calculated by dividing the number of positive cells by the total number of cells.

### Total RNA extraction

Using the Protein and RNA Extraction Kit for mammalian cells (PAREx) (TaKaRa), a cell-lysing solution was prepared, and total RNA was extracted from the recovered chondrocytes, according to the manual of the kit. The extracted total RNA was dissolved with 30 μl of RNA preparation water. The absorbance of RNA was determined using a 50 fold dilution of this solution, and the amount of RNA to be used for RT-PCR was adjusted.

### Reverse transcriptase-polymerase chain reaction (RT-PCR)

sing the One Step RNA PCR Kit (TaKaRa), cDNA was synthesized, and target cDNA was amplified, according to the manual of the kit. PCR was performed using the primers shown in Table 1 with the cycle changes shown below. Concerning TIMP-1, TIMP-2, TIMP-3, and MMP-3, after DNA was denaturated by heating at 94ºC for 2 minutes, the heat denaturation reaction was performed at 94ºC for 45 seconds, annealing reaction was performed at 60ºC for 45 seconds, primer elongation reaction was performed at 72ºC for 1 minute and 30 seconds, and this cycle was repeated 35 times. For glyceraldehyde-3-phosphate dehydrogenase (GAPDH), the heat denaturation reaction was performed at 94ºC for 1 minute, annealing reaction was performed at 60ºC for 1 minute, and primer elongation reaction was performed at 72ºC for 2 minutes, and this cycle was repeated 26 times. In these processes, the final elongation reaction was performed at 72ºC for 10 minutes. The amplified PCR products were identified by electrophoresis using 2% agarose gel prepared by dissolving 0.3 g of agarose (TaKaRa) with 30 ml of TAE (40 mM Tris-acetate 1 mM EDTA, pH7.8) buffer and adding 3 μl of 10 mg/ml ethidium bromide.

**Table 1:** Base sequences of the primers used for RT-PCR

Gene	Base pairs	Primer sequence	Primer source

TIMP-1	326	GCAATCCCGACCTTGTCATC	Reno *et al* (20)
		AGCGTAGGTCTTGGTGAAGC	
TIMP-2	416	GTAGTGATCAGGGCCAAAG	Reno *et al* (20)
		TTCTCTGTGACCCAGTCCAT	
TIMP-3	454	TCTGCAACTCCGACATCGTG	Ishii *et al* (21)
		CGGATGCAGGCGTAGTGTT	
MMP-3	515	AGATGCTGTTGATTCTGCTGTTGAG	Hanke *et al* (22)
		ACAGCATCAAAGGACAAAGCAGGAT	
GAPDH	304	GATGGTGAAGGTCGGAGTGAA	Hanke *et al* (22)
		GGTGAAGACGCCAGTGGATT	

### Evaluation using alginate bead culture medium

**Assay of GAG in the culture supernatant.** The amount of glycosaminoglycan (GAG) in the culture supernatant was determined by the dimethylmethylene blue assay based on the method of Farndale *et al* (23).

**Determination of the amounts of TIMP-1, TIMP-2, and proMMP-3 in the culture supernatant.** The amounts of TIMP-1, TIMP-2, and proMMP-3 in the culture supernatant were determined by the enzyme-linked immunosorbent assay (ELISA) using the methods of Komada *et al* (24) Fujimoto *et al* (25) and Morita *et al* (26) respectively. Concerning TIMP-1 and TIMP-2, the sums of the bound forms present as complexes with MMPs and free forms not forming complexes, i.e., total TIMP-1 and -2 levels, were determined.

### Statistical analyses

Statistical analyses were performed using Student’s t-test at a significance level of *p*<0.05.

## RESULTS

### Percentage of lacunae with no staining for cell-associated matrix (CAM) relative to the total number of lacunae

**Figure 4:**
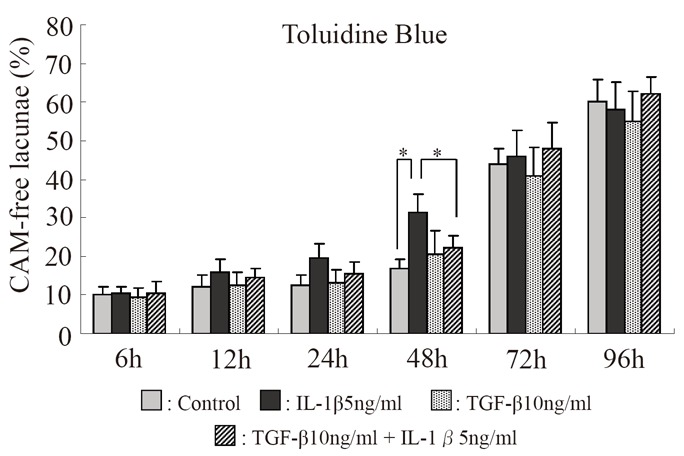
Serial changes in the effects of IL-1β and TGF-β on the percentage of lacunae showing no toluidine blue staining for CAM. *p<0.05, Bar = S.E.

**Figure 5:**
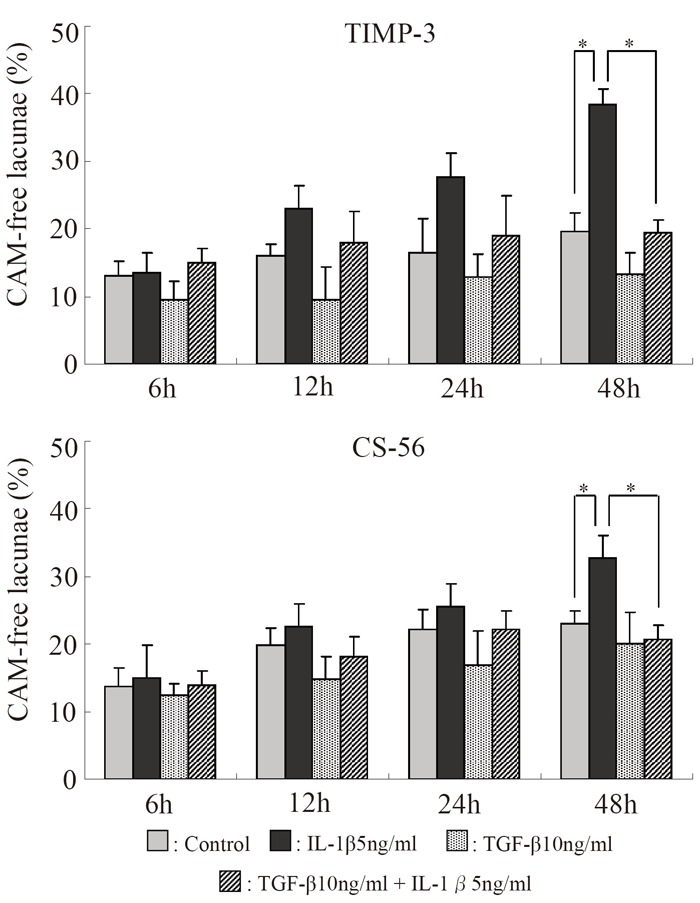
Serial changes in the effects of IL-1β and TGF-β on the percentages of lacunae showing no immunostaining for CAM using anti-TIMP-3 antibody and CS-56. *p<0.05, Bar = S.E.

The percentage of lacunae with no toluidine blue-stained CAM increased progressively in all experimental groups. The percentage of lacunae with no staining for CAM was nearly the same in all groups at 6 hours, but it markedly increased with time in the IL-1β group. In the IL-1β + TGF-β stimulation group, the increase was less marked than in the IL-1β group but was significant after 48 hours. After 72 and 96 hours, the percentage of lacunae with no staining for CAM markedly increased in the control group, and no difference compared with the drug-stimulated groups was noted. Therefore, the duration of evaluation was decided as 48 hours (Fig. 4). The results of immunostaining with anti-TIMP-3 monoclonal antibody and CS-56 were similar to those of toluidine blue staining (Fig. 5).

### Percentages of TIMP-1, TIMP-2, and TIMP-3-positive cells relative to the total number of cells (Fig. 6)

The percentage of TIMP-1-positive cells increased significantly in the IL-1β and IL-1β+TGF-β groups after 6 hours. Thereafter, it decreased with time in the IL-1β group but remained almost unchanged in the IL-1β+TGF-β group. In the TGF-β group, it increased serially, and the increase was significant after 48 hours. The percentage of TIMP-2-positive cells was increased at all points, but the increases were not significant. The percentage of TIMP-3-positive cells was increased after 12 or more hours in both IL-1β and IL-1β+TGF-β groups. Particularly, it increased significantly in the TGF-β group after 24 or more hours and in the IL-1β group after 6 to 48 hours.

**Figure 6:**
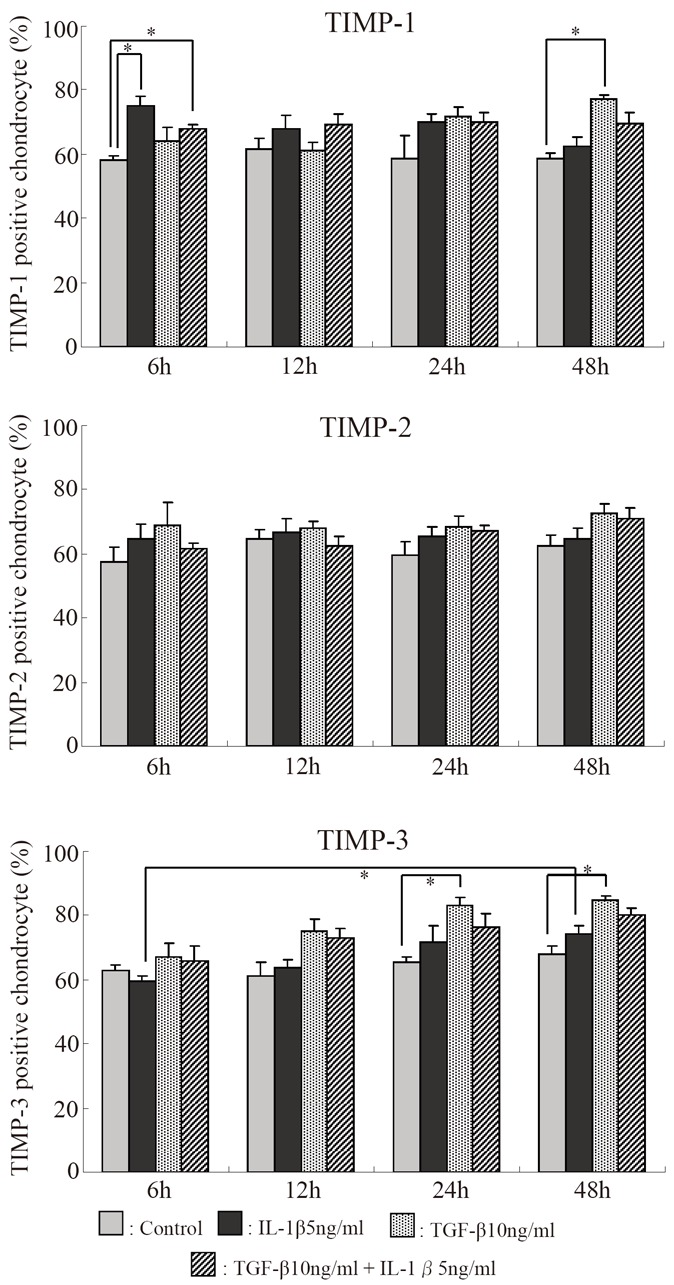
Serial changes in the effects of IL-1β and TGF-β on the percentages of cells stained with anti-TIMP-1, TIMP-2, and TIMP-3 monoclonal antibodies. *p<0.05, Bar = S.E.

### Expression of mRNAs of TIMP-1, TIMP-2, TIMP-3, and MMP-3 (Fig. 7)

The expression of mRNA of TIMP-1 was maximal after 6 hours in the IL-1β group and decreased with time thereafter, but its changes were unrelated to time in the TGF-β and IL-1β+TGF-β groups. The expression of mRNA of TIMP-2 increased slightly at all points in the TGF-β and IL-1β+TGF-β groups, but it was observed only after 24 or more hours in the IL-1β group. The expression of mRNA of TIMP-3 was observed at all points in the TGF-β and IL-1β+TGF-β groups but only after 48 hours in the IL-1β group. However, mRNA of MMP-3 was expressed at all points in the IL-1β and IL-1β+TGF-β groups.

**Figure 7:**
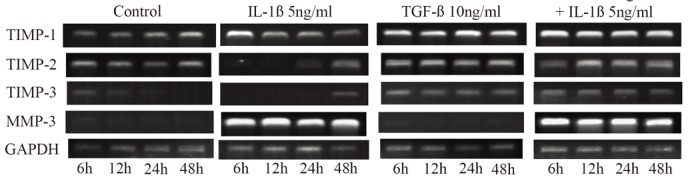
Serial changes in the effects of IL-1β and TGF-β on the expression of mRNAs of TIMP-1, TIMP-2, TIMP-3, and MMP-3.

### Amounts of GAG and proMMP-3 in the culture supernatant (Fig. 8)

The amounts of both GAG and proMMP-3 in the culture supernatant tended to increase with time. Those in the TGF-β group were similar to those in the control group, but they tended to increase in the IL-1β group. In the IL-1β + TGF-β group, however, the IL-1β-induced increases in the amounts of GAG and proMMP-3 tended to be suppressed, and were significantly suppressed after 48 hours.

**Figure 8:**
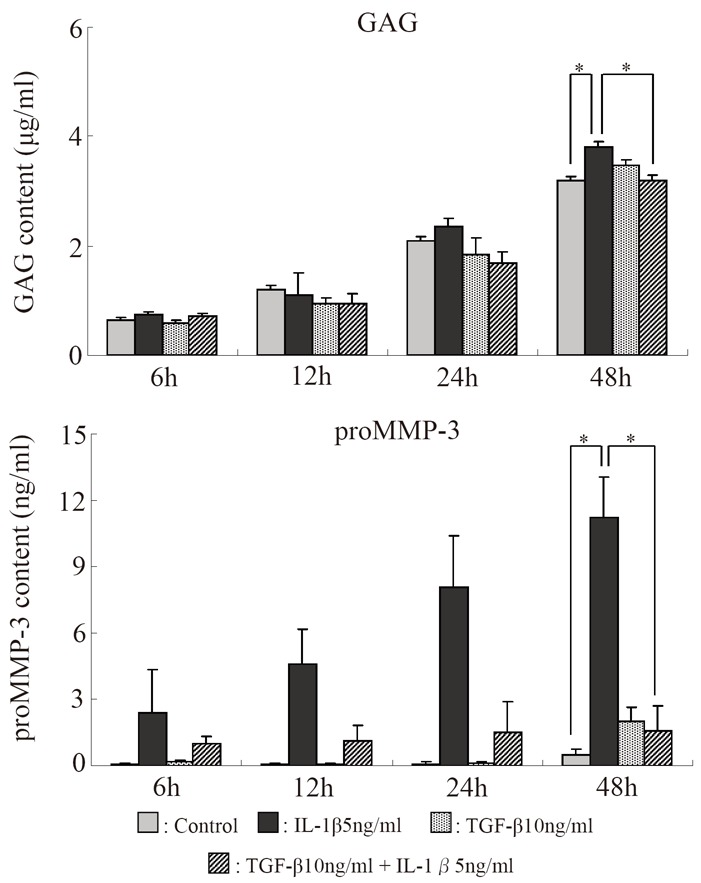
Serial changes in the effects of IL-1β and TGF-β on the amounts of GAG and proMMP-3 in the supernatant of alginate bead cultures. *p<0.05, Bar = S.E.

### Amounts of TIMP-1 and TIMP-2 in the culture supernatant (Fig. 9)

The amount of TIMP-1 in the culture supernatant was maximal after 6 hours in the IL-1β group but decreased with time thereafter, and became significantly lower than in the control group after 48 hours. In the IL-1β+TGF-β group, it was increased after 6 hours, but little decrease was noted thereafter. The amount of TIMP-2 was increased after 6 hours in the IL-1β group, but the increase was not significant.

**Figure 9:**
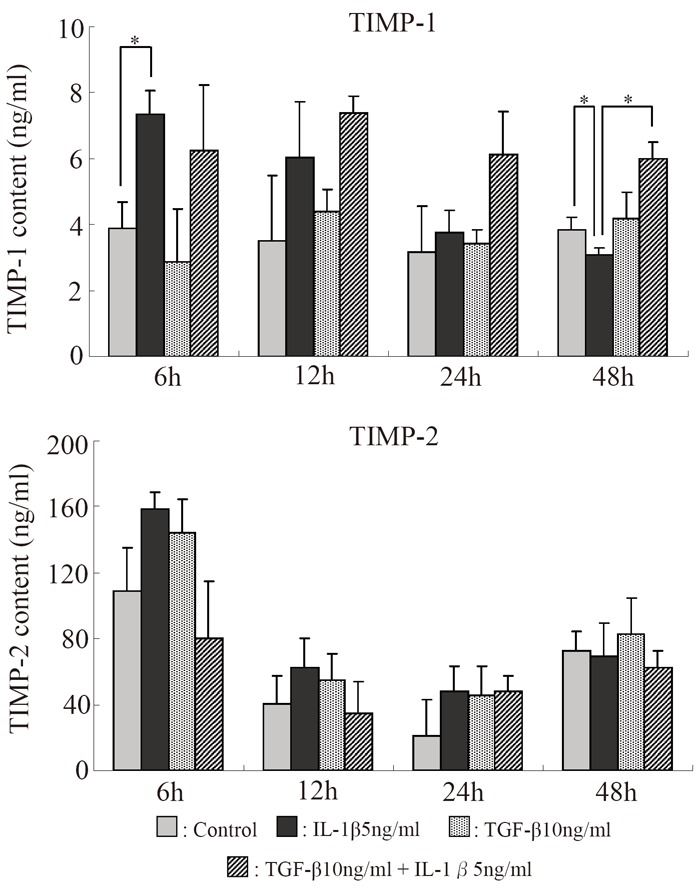
Serial changes in the effects of IL-1β and TGF-β on the amounts of TIMP-1 and TIMP-2 in the supernatant of alginate bead cultures. *p<0.05, Bar = S.E.

## DISCUSSION

Articular cartilage is avascular, relatively simple tissue and consists of chondrocytes and extracellular matrix (ECM). ECM is composed mainly of collagen and proteoglycan, with type II collagen accounting for most of the collagen and aglycan accounting for most of the proteoglycan. Under physiologic conditions, the homeostasis of articular cartilage is maintained by the balance between ECM synthesis and degradation, but this balance is disrupted in joint disorders primarily affecting cartilage such as OA, and the impairment of joint function occurs due to the destruction of ECM components. In osteoarthritic joints, various cytokines are expressed and are involved in both the progression of the disease and its inhibition. Inflammatory cytokines, typically IL-1β, are involved in disease progression (27). IL-1β not only suppresses ECM production but also contributes to its destruction by inducing MMPs and ADAMTS-4 and -5. TGF-β, on the other hand, is a cytokine involved in the inhibition of disease progression (28, 29). It is considered to be a cartilage destruction-preventing factor, because it promotes TIMP production by inhibiting MMPs such as collagenase and stromelysin at the transcription level, as well as promotes cell proliferation and ECM production of articular chondrocytes. These observations have led to evaluations of cartilage destruction using IL-1β or TGF-β. Concerning culturing conditions, Demoor-Fossard *et al* (30) treated chondrocytes in culture with these cytokines, noted differences in ECM formation between monolayer and three-dimensional cultures, and reported that responses to biological stimuli such as cytokines similar to those of cartilage tissue were observed in three-dimensional cultures. This suggests that the responses observed in the ECM in monolayer cultures may not necessarily reflect physiologic responses. In this study, therefore, three-dimensional culturing using alginate beads, which yields results closer to *in vivo* responses, was used.

Since the balance between proteinases and their inhibitory factors is important in the evaluation of cartilage destruction and its inhibition, we directed particular attention to TIMP, a destruction-preventing factor. Four types of TIMP have been reported to date, and they play important roles in the maintenance of ECM integrity as a common endogenous inhibitor of the MMP family, a representative of which is MMP-3 (31). However, much remains unclear about which TIMPs play roles in the inhibition of ECM destruction or at what timing they act. In this study, therefore, we prepared an inflammation model by IL-1β stimulation and serially evaluated changes.

First, concerning the degree of destruction due to IL-1β stimulation, the percentage of lacunae without CAM tended to increase with time. The amounts of GAG and proMMP-3 in the culture supernatant also increased with time, and mRNA of MMP-3 was expressed at all points of evaluation. These results illustrated the progression of CAM degradation associated with increases in the expression of MMP-3 under IL-1β stimulation. On the other hand, when this inflammation model was simultaneously treated with TGF-β, the increases in the percentage of lacunae lacking CAM and the amounts of GAG and proMMP-3 in the culture supernatant observed under IL-1β simulation were suppressed to the control levels after 48 hours. These results established that TGF-β has a protective effect against CAM destruction. However, the mRNA level of MMP-3 was not affected by TGF-β at any point of evaluation. Therefore, MMP-3 is considered to be secreted under simultaneous stimulation with IL-1β and TGF-β as under stimulation with IL-1β alone, but TIMP is considered to increase serially under simultaneous stimulation and inhibit CAM destruction by binding with active MMP including MMP-3.

Next, regarding serial changes in the TIMP level, changes in the TIMP-1 and TIMP-3 levels under IL-1β simulation differed. Concerning TIMP-1, the positive cell rate and amount in the culture supernatant peaked after 6 hours and decreased serially thereafter. The mRNA level also showed similar changes and was reduced after 48 hours. Concerning TIMP-3, in contrast, the positive cell rate and mRNA level showed reverse changes compared with TIMP-1. These results suggest that chondrocytes produce and secrete a large amount of TIMP-1, and that TIMP-1 plays a primary role in the prevention of CAM destruction against MMP in an early stage of inflammation, but that TIMP-3 gradually takes over this role as the primary preventive factor against CAM destruction. However, in the group simultaneously treated with IL-1β and TGF-β, the expression of TIMP-1 was maintained throughout the evaluation period. Regarding TIMP-3, the expression of mRNA was increased at all evaluation points, and the positive cell rate was higher than in the IL-1β group. These results suggest that TIMP-1 and TIMP-3 are closely involved in the inhibition of cartilage destruction by TGF-β, and that TGF-β prevents cartilage destruction by enhancing the expression of TIMP-1 and TIMP-3 from an early stage. Regarding TIMP-2, no marked change was noted under either stimulation. This suggests that TIMP-2 functions in the body as a protein that always exists in cells at a fixed level (32).

Kevorkian *et al* (33) reported the expression of mRNAs of TIMPs in cartilage tissue of OA patients compared with normal human articular cartilage as follows: No change in the expression of TIMP-2 was noted, the expression of TIMP-1 and TIMP-4 was reduced, but that of TIMP-3 was increased, suggesting that the decreases in the expression of TIMP-1 and TIMP-4 may have important implications in the process of cartilage matrix destruction due to an imbalance between proteinases and their inhibitors. While TIMP-4 was not evaluated in this study, as the expression of TIMP-1, TIMP-2, and TIMP-3 mRNAs after 48 hour stimulation with IL-1β was in agreement with their findings, the results after 48 hours are considered to closely reflect the pathology of OA. The change in the TIMP-1 level after 48-hour treatment with IL-1β and TGF-β is reflected in not only the mRNA level but also its release into the culture medium, and this suggests, as mentioned above, that TIMP-1 is the primary TIMP that binds to active MMP including MMP-3.

TIMP-3, on the other hand, has been shown to bind specifically with sulfated GAG such as chondroitin sulfate via its N-terminal (10). In this study, also, the percentages of lacunae lacking CAM showed similar changes on immunostaining for TIMP-3 and chondroitin sulfate. Therefore, TIMP-3 bound with the ECM is considered to act as a barrier for the ECM and to protect it from degradation by MMP. In addition, TIMP-3 is known to inhibit not only MMP but also aggrecanase, which is considered to be another major ECM-degrading enzyme (11). Since the expression of both aggrecanases-1 and -2 is increased in OA cartilage (34), TIMP-3, which is the only inhibitor of these enzymes, may play a greater role in the inhibition of joint destruction than TIMP-1 or TIMP-2.

## CONCLUSION

The results of this study suggest that TIMP-1 plays a primary role in the prevention of articular cartilage destruction in its early stage but that TIMP-3 gradually takes over this role. Also, TGF-β was shown to regulate these TIMPs and act as a suppressor of articular cartilage destruction. These results suggest that TIMP-1 and TIMP-3 are closely involved in preventing the progression of joint disorders such as OA.
